# Acromegaly and Morris Syndrome: Description of a Clinical Case

**DOI:** 10.2174/0118715303424098250925063845

**Published:** 2025-10-16

**Authors:** Simone Antonio De Sanctis, Sabrina Chiloiro, Antonella Giampietro, Angelo Minucci, Liverana Lauretti, Marco Gessi, Guido Rindi, Alessandro Olivi, Laura De Marinis, Francesco Doglietto, Antonio Bianchi, Alfredo Pontecorvi, Ettore Domenico Capoluongo

**Affiliations:** 1 Fondazione Policlinico Universitario A. Gemelli IRCCS, UOC Endocrinologia e Diabetologia, Rome, Italy;; 2 Fondazione Policlinico Universitario A. Gemelli IRCCS, Dipartimento di Medicina Traslazionale, Rome, Italy;; 3 Departmental Unit of Molecular and Genomic Diagnostics, Fondazione Policlinico Universitario A. Gemelli IRCCS, 00168 Rome, Italy;; 4 Fondazione Policlinico Universitario A. Gemelli IRCCS, Neurosurgery, Rome, Italy;; 5 Fondazione Policlinico Universitario A. Gemelli IRCCS, Department of Life Sciences and Public Health, Section of Anatomic Pathology, Rome, Italy;; 6 Department of Molecular Medicine and Medical Biotechnologies, University of Naples Federico II, Naples, Italy

**Keywords:** Acromegaly, pituitary adenoma, growth factor-I (IGF-I), morris, androgen, hypertension

## Abstract

**Background:**

Acromegaly associated with Morris syndrome has never been reported in the literature.

**Case Presentation:**

We present the case of a 49-year-old woman with Morris syndrome, diagnosed in 1992, who has undergone gonadectomy and hormone replacement therapy for about 15 years. The patient was referred to our centre for the clinical suspicion of acromegaly in June 2022, for the enlargement of the acral extremities and the development of prognathism in the last 10 years. The patient underwent mandibular reduction surgery and removal of a tubular adenoma of the colon in 2010. In June 2021, the patient performed random GH, IGF-I, and prolactin (PRL) dosages that confirmed the diagnosis of acromegaly. A contrasted pituitary MRI showed the presence of an 8 mm intrasellar pituitary adenoma. Therefore, a transsphenoidal resection of the pituitary tumor was conducted in September 2021. The histological examination proved the diagnosis of somatotropinoma. At the last follow-up at our center in June 2024, the patient presented in a fair general clinical condition, with recovery of related acromegaly symptoms, normalized IGF-I levels, and a negative pituitary MRI for signs of somatotropinoma recurrence.

**Conclusion:**

Our clinical case describes for the first time the association between Morris syndrome and acromegaly. Due to the singularity of this case, we decided to conduct more in-depth genetic analyses through a clinical exome study and CGH Array evaluation, which, however, did not lead to the discovery of a genetic association between the two conditions.

Although this condition is rare, further genetic studies are needed to demonstrate a genetic association between these two conditions.

## 1 INTRODUCTION

Acromegaly is a rare disease related to excessive production of growth hormone (GH) by a pituitary adenoma/neuroendocrine tumour, and consequently increased liver synthesis of insulin-like growth factor-I (IGF-I) [1]. Patients with acromegaly develop a typical symptomatology, including elongation of the jaw, prominent frontal bumps, protruding cheekbones, prognathism, increased size of the nose and ears (so-called ‘acromegalic facies’), bone deformity with enlargement of the extremities, peripheral arthropathy and osteoporosis, hypertension, cardiomyopathies and valvopathies, increased susceptibility to tumours (especially of the colon and thyroid), hyperglycaemia, type II diabetes mellitus, and weight gain [2-7].

Acromegaly may rarely occur in the context of genetically determined syndromes associated with germline or somatic mutation of genes located on several chromosomes [1].

Acro-gigantism has been reported to be associated with a microduplication of the GRP101 gene, located in the Xq26.3 chromosomal region, which is responsible for a form of early-onset acro-gigantism (X-LAG), with some variability in somatic mosaicism patterns between the two sexes [1]. In fact, GRP101-related X-LAG is clinically and genetically distinct from adult-onset acromegaly.

Until now, no case of concomitant acromegaly and Morris syndrome has been reported. Morris syndrome (or androgen insensitivity syndrome) is a rare X-linked disorder in which patients with XY chromosomes, corresponding to a male genotype, develop female sexual characteristics. Morris syndrome represents the most common cause of disorders of sexual differentiation in individuals with a 46, XY karyotype. The phenotype of the disease is variable, with complete (CAIS), mild (MAIS), and partial forms (PAIS). In CAIS, patients typically show normal female external genitalia; in MAIS, patients typically show normal male external genitalia but suffer from infertility and/or gynaecomastia. Instead, PAIS is defined in patients who have underdeveloped male external genitalia. PAIS should be considered in all individuals with ambiguous genitalia at birth, irrespective of the degree of virilisation, whereas MAIS should be suspected in males with persistent gynaecomastia and/or infertility that cannot otherwise be explained [8, 9].

The diagnosis of Morris syndrome is, therefore, based on clinical suspicion and confirmed by genetic analysis of the karyotype. The phenotypic heterogeneity of the disease depends on the type of mutation affecting the androgen receptor (AR) gene, which is in the Xq11-12 chromosomal region. Early diagnosis and appropriate management of this disease are crucial to prevent short- and long-term complications and provide adequate psychological support to patients [8, 9].

In this case report, we described the clinical history of a patient affected by acromegaly and Morris syndrome.

## 2 CASE REPORT

A 49-year-old woman with a history of Morris syndrome (CAIS) was referred to our Pituitary Unit in June 2021 for progressive enlargement of acral extremities, which occurred in the previous 10 years. The clinical case history is shown in Table **1**.

## 3 DIAGNOSIS AND MANAGEMENT OF MORRIS SYNDROME

### 3.1 Diagnosis

In 1992, when the patient was 19 years old, Morris syndrome was diagnosed, for the primary amenorrhea, underdeveloped breasts, abnormal height, and presence of an inguinal hernia. Genetic analyses identified the AR homozygote c.1048C>T mutation.

### 3.2 Treatment

The patient underwent gonadectomy to prevent a possible malignant transformation of the testicular tissue situated at the abdominal level, and subsequently, hormone replacement therapy for primary amenorrhoea was started.

### 3.3 Diagnosis and Management of Acromegaly

#### 3.3.1 Diagnosis

In 2020, the patient had also undergone mandibular reduction surgery and the removal of a tubular adenoma of the colon. The patient also reported the following in her previous clinical history: multinodular thyroid goiter, primary autoimmune hypothyroidism in treatment with levothyroxine, hypovitaminosis D in replacement therapy with cholecalciferol, arterial hypertension in treatment with beta-blockers and thiazide diuretic, generalised anxiety disorder in treatment with benzodiazepines, and grade III obesity (body mass index: 40.7).

The patient’s family history did not include endocrine diseases or pituitary tumors.

**Fig. (1):**
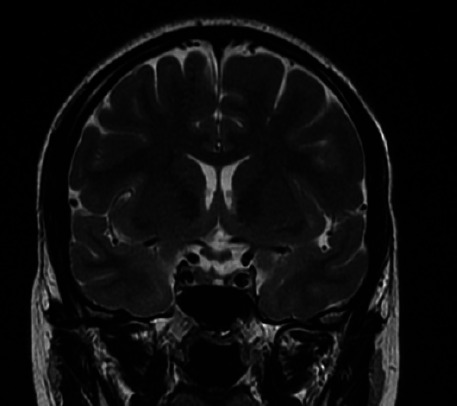
Microadenoma.

At our Pituitary Unit, pituitary hormone was tested in the clinical suspicion of acromegaly, providing high levels of GH (7.14 mcg/L) and IGF-I (686 ng/mL, while the normal range for the 41-50 age group is 90-300 ng/mL), in the absence of hyperprolactinemia and hypopituitarism. A pituitary magnetic resonance imaging with contrast medium showed the presence of a right latero-basal pituitary adenoma, with ill-defined margins, ovoid in shape, around 8 mm in diameter, with infero-posterior development, which exerted an erosive effect on the right half of the floor, near the sellar dorsum (Fig. **1**).

### 3.4 Treatment

A transsphenoidal resection of the pituitary tumour was performed in September 2021. The histological examination proved a densely granulated somatotroph tumor, with multifocal and intense expression of the GH and of Pit-1, with proliferative index (Mib-1) of around 1%, with positivity for p53 in around 10-15% of cells, and with intense and membranous positivity for the subtype 2 of the somatostatin receptor (SSTR2A).

### 3.5 Follow-up

During a 3-year follow-up, no symptoms of active acromegaly were observed, IGF-I levels remained within reference ranges, and the pituitary MRI did not show signs of recurrent disease. The screening for acromegaly-related comorbidities showed a multinodular thyroid goiter, liver steatosis, and mild aortic ectasia, and excluded the presence of colon of pre-cancerous and carcinomatous lesions.

According to the concomitant diagnosis of Morris syndrome and acromegaly, we decided, after collecting informed consent from the patient, to deeply investigate genetic abnormalities in our patient. For this purpose, two complementary approaches were used: the CGH (Comparative Genomic Hybridization) array and clinical exome analysis, both performed on the patient’s blood sample.

However, the results were inconclusive, leaving open the possibility of a yet unidentified genetic cause undetectable by these approaches to link the two conditions described above.

In fact, the clinical exome analysis performed using the massive parallel sequencing (NGS) technique on the coding plus exon-intron junction regions of 4800 genes (including, in particular, ABCC9, AR, AIP, MEN1, GNAS and PRKAR1A), exclusively confirmed the already formulated diagnosis of Morris syndrome, confirming the homozygosity of the AR c.1048C>T mutation (rs137852562). No genetic variants of uncertain significance (VUS) were observed. On the other hand, genomic analysis by array-CGH did not reveal microdeletions and/or microduplications in the autosomes and sex chromosomes associated with the indicated clinical phenotype. Incidental alterations not directly attributable to the clinical phenotype that were nevertheless described by our analysis include the following:

- an interstitial microdeletion of the short arm of a chromosome 16, in the region 16p13.3, extending approximately 14 Kb (arr(GRCh37) 16p13. 3(215724_229956)x1) and involving the OMIM disease genes HBA1 (*141800) and HBA2 (*141850) and the OMIM gene HBM (*609639); this rearrangement overlaps with the -CANT microdeletion associated with alpha thalassaemia (#604131): due to the finding of this mutation, the patient was referred for haematological follow-up;
- an interstitial microdeletion of the short arm of a chromosome 16, in the region 16p13. 3, extending approximately 75 Kb (arr[GRCh37] 16p13.3(6929568_ 7004381)x1), intragenic to the OMIM gene RBFOX1 (*605104); RBFOX1 gene rearrangements are reported in scientific literature in association with neurodevelopmental disorders with incomplete penetrance and variable expressivity.

## 4 DISCUSSION

In this study, we report the clinical history of a patient affected by acromegaly and Morris syndrome, both of which are recognized as rare diseases.

Acromegaly can occur in the context of a genetically determined syndrome, due to germline or somatic mutations. Germline GNAS mutations are associated with McCune-Albright syndrome, and somatic mutations are present in approximately 40% of GH-secreting tumours. Germline mutations are also reported, including respectively the mutation of the gene AIP (which is associated with the FIPA, familiary isolated pituitary adenoma), the MENIN gene (which is associated with the MEN-1 syndrome), the CDKN1B gene (which is associated with the MEN-4 syndrome), the PRKAR1A gene (which is associated with the Carney complex syndrome), the SDHx gene (which is associated with the acromegaly and paragangliomas/ phaeochromocytoma, namely 3 PAs syndrome), the RET gene (which is associated with the xxx syndrome) and the GPR101 gene (which is associated with the X-linked acro-gigantism) [10-16]. The microduplications of the GPR101 gene in the Xq26.3 region have been identified as the cause of X-LAG syndrome [14-16]. GPR101 protein is primarily expressed in the brain, particularly in the pituitary gland, and is involved in regulating energy balance, cell reproduction, and migration. Patients usually carry pituitary adenomas more frequently, which are mixed and secrete both GH and PRL, with variable expression of somatostatin receptors. Although the penetrance of the disease is 100%, most patients with X-LAG syndrome present the disease in sporadic form (72.2%), probably due to the occurrence of de novo mutations, as in Morris syndrome. Tumours in X-LAG syndrome are usually macroadenomas (75% of cases) with suprasellar extension and an invasive growth pattern; in contrast, 25% of patients with X-LAG syndrome have pituitary hyperplasia. Recently, other mutations in the GPR101 gene (p.E308D and p.D366E) have been identified in patients with sporadic forms of acromegaly in adulthood, suggesting that other GPR101 variants may contribute to the pathogenesis of the disease [10, 11, 15, 16].

However, it is worth noting that X-LAG is a rare, early-onset gigantism distinct from adult-onset acromegaly, both in terms of pathophysiology and clinical course. The current case does not meet clinical or molecular criteria for X-LAG. No duplication or mutation of GPR101, or other acromegaly-related genes (*e.g*., GNAS, AIP), was identified in this patient. Thus, the available data do not support a shared etiological basis between CAIS and acromegaly in this case. Moreover, no pathogenic variants or chromosomal rearrangements related to GH excess or pituitary adenomas were identified. The two incidental findings involving 16p13.3 (microdeletions of HBA1/HBA2 and RBFOX1) are unrelated to either CAIS or acromegaly, without supporting a direct genetic link between CAIS and acromegaly. Therefore, the co-occurrence of these rare diseases is more likely coincidental, given the known frequency of de novo mutations in both conditions.

Interestingly, the AR gene is located on chromosome Xq11-12 and consists of eight exons and codes for a protein of 919 amino acids [8]. Morris syndrome is caused by missense mutations, resulting in an amino acid change, which can potentially affect each of the eight exons of the AR gene [9]. The mutations discovered so far are numerous, and the total number reported has been steadily increasing in recent years, rising from 76 to 159 between 2004 and 2012, as described in a report by Gottlieb and colleagues [17]. In approximately 30-40% of cases, however, there is no family history of Morris syndrome, suggesting that many of the mutations causing Morris syndrome may arise de novo [8, 18, 19].

To date, no cases have been reported in which Morris syndrome was associated with acromegaly. However, a link between these conditions may be speculated, taking into account that syndromic acromegaly can be due to microduplications of the GPR101 gene in the Xq26.3 region, and that Morris syndrome is due to a mutation of the AR gene, which is located on the X chromosome.

In this clinical case, we performed both CGH Array and clinical exome analysis. The CGH Array is an advanced technique that makes it possible to detect small variations in the copy number of DNA segments (in particular deletions and duplications, as occurs in the cases described in the literature of X-LAG) over the entire genome, thus including the X and Y sex chromosomes [20-22]. Clinical exome, on the other hand, involves the analysis of the genome coding regions (or exons) and is used to identify especially point mutations (including those responsible for Morris syndrome), allowing areas of interest to be sequenced at a high resolution [23]. With the above methods, we only confirmed the presence of the homozygous c.1048C>T AR gene mutation, but we found no mutations potentially associated with acromegaly.

The fact that a patient simultaneously presents with two rare endocrinopathies, such as Morris syndrome and acromegaly, raises several issues related to clinical management. In fact, although we failed to identify a univocal genetic alteration, some similarities among these two diseases can be identified.

Morris syndrome predisposes to an increased risk of gonadal and vulvo-vaginal tract neoplasms, including mainly gonadoblastomas and seminomas, and squamous cell carcinoma of the vulva and of the vagina [24-26]. Acromegaly, instead, is classically associated with an increased risk of cancers in general. Although there is no data available for gonadal cancers, one can easily conclude that a patient with both conditions can have a higher overall neoplastic risk [2, 5].

The neuropsychological profile is typically impaired in Morris syndrome and in acromegaly. The patient that we are reporting was affected by generalised anxiety and was under treatment with benzodiazepine. Moreover, around one third of acromegalic patients show symptoms of major depression and, recently, two large Italian multicentre studies using neurocognitive and neuropsychological questionnaires also reported comparable rates of depressive symptoms (between 28% and 30%) in patients with acromegaly, with a significant percentage of these also presenting with severe symptoms requiring drug therapy [27, 28].

Finally, there is no certain data in the literature regarding the prevalence of the association between Morris syndrome and psychological or psychiatric disorders.

In light of all this, the multidisciplinary management of a patient with both acromegaly and Morris syndrome is essential to ensure a complete and integrated treatment, addressing both endocrinological and genetic, psychological and functional aspects, to improve quality of life and prevent long-term complications [29-33].

## 5 LIMITATIONS

The limitations of this study are related to the fact that there are no other reports on the coexistence of acromegaly and Morris syndrome and genetic studies that investigated a potential association. In this way, our report may also not support a genetic link between Morris syndrome and acromegaly, which is often assumed in X-linked acromegaly disease.

## CONCLUSION

This clinical case describes, to our knowledge, for the first time, the clinical association between Morris syndrome and acromegaly.

As these are very rare conditions, further genetic studies are needed to demonstrate a genetic link. In fact, our genetic work-up yielded no novel or unifying mechanism between the two diseases, reinforcing the interpretation of a coincidental co-occurrence.

Furthermore, a multidisciplinary management is advocated for these patients, for the diagnosis and treatment of disease-related complications, mainly an impaired neuropsychological profile.

## Figures and Tables

**Table 1:** Clinical case history.

January 1992	First endocrinological examination at the age of 19 for: primary amenorrhea, underdeveloped breasts, abnormal height, presence of inguinal hernia
November 1992	Genetic analyses: AR homozygote c.1048C>T mutation
December 1992	Gonadectomy
1993 - 2010	Hormone replacement therapy
March 2020	Mandibular reduction surgery
June 2020	Removal of a tubular adenoma of the colon
June 2021	GH 7.14 mcg/L and IGF-I 686 ng/mL
July 2021	A pituitary magnetic resonance imaging with contrast medium showed the presence of a right latero-basal pituitary adenoma around 8 mm
September 2021	Transsphenoidal resection of the pituitary tumour
January 2025	Last follow-up: no symptoms of active acromegaly occurred, IGF-I levels remained within reference ranges and pituitary MRI did not show signs of recurrent disease

## Data Availability

All the data and supporting information are provided within the article.

## LIST OF ABBREVIATIONS

PRL: Prolactin
GH: Growth Hormone
IGF-I: Insulin-Like Growth Factor-I
AR: Androgen Receptor
CGH: Comparative Genomic Hybridization
VUS: Variants Of Uncertain Significance

## STANDARDS OF REPORTING

CARE guidelines were followed.
